# Potential Roles of Enterochromaffin Cells in Early Life Stress-Induced Irritable Bowel Syndrome

**DOI:** 10.3389/fncel.2022.837166

**Published:** 2022-03-15

**Authors:** Enfu Tao, Zhenya Zhu, Chenmin Hu, Gao Long, Bo Chen, Rui Guo, Marong Fang, Mizu Jiang

**Affiliations:** ^1^Endoscopy Center and Gastrointestinal Laboratory, Children’s Hospital, Zhejiang University School of Medicine, National Clinical Research Center for Child Health, National Children’s Regional Medical Center, Hangzhou, China; ^2^Wenling Maternal and Child Health Care Hospital, Wenling, China; ^3^Institute of Neuroscience and Gastrointestinal Laboratory, Children’s Hospital, Zhejiang University School of Medicine, Hangzhou, China; ^4^Department of Gastroenterology, Children’s Hospital, Zhejiang University School of Medicine, National Clinical Research Center for Child Health, National Children’s Regional Medical Center, Hangzhou, China

**Keywords:** enterochromaffin cells (ECs), irritable bowel syndrome (IBS), early life stress (ELS), brain–gut–microbiota axis, 5-hydroxytryptamine

## Abstract

Irritable bowel syndrome (IBS) is one of the most common functional gastrointestinal disorders, also known as disorders of the gut–brain interaction; however, the pathophysiology of IBS remains unclear. Early life stress (ELS) is one of the most common risk factors for IBS development. However, the molecular mechanisms by which ELS induces IBS remain unclear. Enterochromaffin cells (ECs), as a prime source of peripheral serotonin (5-HT), play a pivotal role in intestinal motility, secretion, proinflammatory and anti-inflammatory effects, and visceral sensation. ECs can sense various stimuli and microbiota metabolites such as short-chain fatty acids (SCFAs) and secondary bile acids. ECs can sense the luminal environment and transmit signals to the brain via exogenous vagal and spinal nerve afferents. Increasing evidence suggests that an ECs-5-HT signaling imbalance plays a crucial role in the pathogenesis of ELS-induced IBS. A recent study using a maternal separation (MS) animal model mimicking ELS showed that MS induced expansion of intestinal stem cells and their differentiation toward secretory lineages, including ECs, leading to ECs hyperplasia, increased 5-HT production, and visceral hyperalgesia. This suggests that ELS-induced IBS may be associated with increased ECs-5-HT signaling. Furthermore, ECs are closely related to corticotropin-releasing hormone, mast cells, neuron growth factor, bile acids, and SCFAs, all of which contribute to the pathogenesis of IBS. Collectively, ECs may play a role in the pathogenesis of ELS-induced IBS. Therefore, this review summarizes the physiological function of ECs and focuses on their potential role in the pathogenesis of IBS based on clinical and pre-clinical evidence.

## Introduction

Enterochromaffin cells (ECs) are the most abundant type of enteroendocrine cells throughout the gastrointestinal (GI) tract (Lund et al., 2018). They account for only 1% of enteric epithelial cells, however, they secrete 90% of serotonin (5-hydroxytryptamine or 5-HT) in the body (Mawe and Hoffman, 2013). Considering 5-HT content in the gut alone, approximately 95% is synthesized in ECs, whereas 5% is synthesized in enteric neurons and other cell types (Banskota et al., 2019). The rate-limiting enzyme for 5-HT biosynthesis is tryptophan hydroxylase (TPH), which includes TPH1 in ECs and TPH2 in neurons of the enteric nervous system (ENS) and central nervous systems (CNS) (Walther et al., 2003).

ECs are located on the intestinal mucosal surface and the epithelial layer (Mawe and Hoffman, 2013). 5-HT, as a paracrine modulator produced, stored, and released from ECs, is delivered to neighboring cells and neurons (Wood, 2020) and exerts its function through various 5-HT receptors (5-HTR) (De Deurwaerdère et al., 2020). 5-HT is an important neurotransmitter in the CNS and ENS and plays a key role in regulating visceral sensations and emotions, such as anxiety and depression. There are seven 5-HTR (5-HT_1–7_R), and at least 14 subtypes are widely distributed in the intestine and brain (De Deurwaerdère et al., 2020). ECs link epithelial cells, goblet cells (Wood, 2020), afferent vagal neurons (Bellono et al., 2017), and myenteric neurons in the mucosa (Neunlist et al., 1999), and also link the ENS, spinal afferent terminals (Wood, 2020), and enteric mast cells (Wang et al., 2013) in the lamina propria through the interaction of 5-HT and 5-HTR.

Importantly, ECs are the major enteroendocrine cells (Kim and Camilleri, 2000) that can synthesize and secrete various signaling molecules and hormones, such as 5-HT, corticotropin-releasing hormone (CRH) (Kawahito et al., 1994), cholecystokinin (Fakhry et al., 2017), glucagon-like peptide-1 (GLP-1) (Lee et al., 2012), peptide YY (Reynaud et al., 2016), and substance P (SP) (Nøhr et al., 2013). With the endocrine potential, ECs are involved in modulating GI motility and metabolic disorders (Martin et al., 2017b). Furthermore, ECs contain various receptors, which are used by ECs to sense nutritive metabolites, such as glucose, fructose, amino acids, lipid amides, oleoylethanolamides (Martin et al., 2017a), ketones, niacin, aromatic acids, acyl-amides, and lactate (Lund et al., 2018), and microbial metabolites, such as short-chain fatty acids (SCFAs) (Martin et al., 2017a) and secondary bile acids (SBAs) (Lund et al., 2018). Moreover, ECs can sense mechanical forces with the mechanosensitive ion channel Piezo2 to synthesize 5-HT (Alcaino et al., 2018). More recently, ECs have been reported to sense fecal single strand RNA with the mechanosensitive ion channel Piezo1, which is essential for systemic 5-HT synthesis (Sugisawa et al., 2020). Accordingly, ECs act as intestinal sentinel cells and play an important role in regulating intestinal homeostasis. They can sense the luminal environment and transmit signals to the brain via exogenous vagal and spinal nerve afferents. Likewise, signals from the brain can be transmitted to the ECs through the vagus nerve and spinal cord. ECs may play an essential role in the brain–gut–microbiota axis.

Irritable bowel syndrome (IBS) is the most common functional GI disorder, also known as disorders of the gut–brain interaction (Vasant et al., 2021). It is characterized by recurrent episodes of abdominal pain/discomfort and changes in bowel movements, with high morbidity in adults (Enck et al., 2016) and children worldwide (Korterink et al., 2015), resulting in a considerable disease burden and severely affecting the physical and mental well-being and quality of life of patients. Unfortunately, the etiology of IBS remains unclear (Chong et al., 2019). However, brain–gut–microbiota interaction disorder, visceral hypersensitivity, increased intestinal permeability, intestinal motility dysfunction, immune activation, low-grade inflammation, and somatic and psychiatric comorbidities are involved in the pathogenesis of IBS (Enck et al., 2016).

Early life stress (ELS) and its role in the pathogenesis of IBS have gradually attracted increasing attention (Ju et al., 2020). ELS is a risk factor for IBS development (Jones et al., 2020; Ju et al., 2020; Low et al., 2020), and is more common in IBS patients than in healthy controls (Bradford et al., 2012). Furthermore, ELS is correlated with the severity of IBS symptoms (Park et al., 2016). However, the precise molecular mechanisms by which ELS induces IBS remain unclear, and dysbiosis in the composition of commensal bacterial communities, increased intestinal permeability, irregular intestinal motility, visceral hypersensitivity, and anxiety/depression-like behaviors may be implicated (Labus et al., 2017; Fukui et al., 2018; Cojocariu et al., 2020; Sugiyama and Shiotani, 2020; Lingpeng et al., 2021). Recently, studies have shown that ELS increases susceptibility to IBS in later life (Wong et al., 2019; Low et al., 2020), which is associated with an increased number of ECs (Wong et al., 2019). Increasing evidence indicates that ECs play a crucial role in the pathogenesis of ELS-induced IBS (Chow et al., 2019; Qin et al., 2019a; Wong et al., 2019), as ECs bidirectionally (top-down or down-top) mediate signaling transmission of the brain–gut–microbiota axis (Stasi et al., 2012).

Considering the unique features of ECs, this review aims to investigate the pathogenesis of ELS-induced IBS from a new perspective, namely ECs, and hopes to provide novel insights into future in-depth studies and explore new therapeutic strategies for IBS.

## Physiology of Enterochromaffin Cells Origin and Location of Enterochromaffin Cells in the Gut

Intestinal epithelial cells originate from multipotent intestinal stem cells (ISCs). ISCs are differentiated into four distinct intestinal cell types, namely enterocytes, goblet, enteroendocrine, and Paneth cells, by the interaction of wingless/integrated (Wnt), Notch, and bone morphogenetic protein signaling pathways (Smith et al., 2017). ECs are a major intestinal enteroendocrine cell type. They mature and migrate up the villous tips where they are eventually extruded (Schonhoff et al., 2004). Generally, normal ECs are terminally differentiated and non-proliferating. However, ECs are constantly being renewed. The turnover rate of ECs varies substantially, ranging from 16 (approximately 60–65%) to 150 days (35–40%), which is considerably slower than that of the surrounding enterocytes (de Bruïne et al., 1992).

**FIGURE 1:**
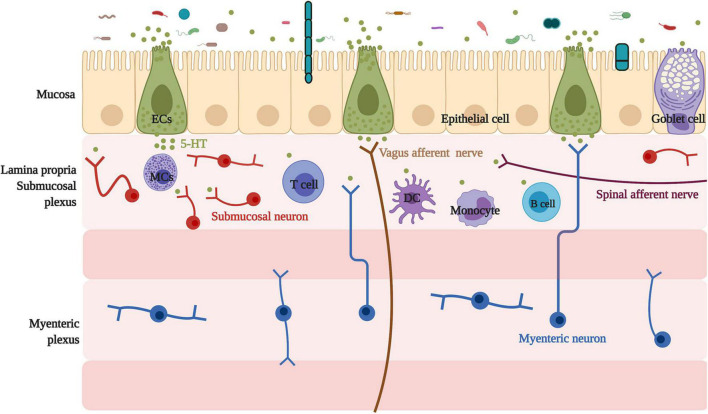
Location of enterochromaffin cells and their association with neighbor cells and neurons. Located on the intestinal mucosal surface, the epithelial layer, ECs have a relationship with neighboring cells, including epithelial and goblet cells, and connect with the submucosal neuron, the myenteric neuron projecting into the submucosal, and nerve endings of the vagal and spinal afferents. In addition, ECs are linked with various immune cells, such as mast cells, monocytes, dendritic cells, T cells, and B cells. ECs, enterochromaffin cells; 5-HT, 5-hydroxytryptophan/serotonin; MCs, mast cells; DC, dendritic cells.

ECs are located on the intestinal mucosal surface, the epithelial layer. Additionally, ECs connect with the nerve endings of the vagal and spinal afferents (Mawe and Hoffman, 2013). Locally, in the lamina propria, ECs-derived 5-HT acts as a major paracrine signal through G protein-coupled 5-HTR on neighboring cells to affect epithelial growth (Tackett et al., 2017), enterocyte secretion, and intestinal barrier function (Akiba et al., 2017), as well as to activate immune cells (Margolis et al., 2014) and enteric nerves (Mawe and Hoffman, 2013). Location of enterochromaffin cells and their association with neighboring cells and neurons are summarized in Figure 1.

## Receptors on Enterochromaffin Cells

ECs contain various receptors, which are useful for sensing both nutritive metabolites, such as glucose, fructose, amino acids, lipid amides, oleoylethanolamides (Martin et al., 2017a), ketones, niacin, aromatic acids, acyl-amides, lactate (Lund et al., 2018) and microbiota generated metabolites, such as SCFAs (Fukumoto et al., 2003; Nøhr et al., 2013; Akiba et al., 2015; Martin et al., 2017a), and SBAs (Alemi et al., 2013; Lund et al., 2018). Importantly, free fatty acid receptor 2 (FFAR2) and FFAR3 (Martin et al., 2017a) of ECs can directly sense SCFAs in the intestinal tract and provide the media for the interactions of the brain–gut–microbiota axis (Dalile et al., 2019). However, receptors expressed on ECs differ in the intestine. For example, ECs from the small intestine do not express G protein-coupled receptor (GPCR) sensors for lipid and protein metabolites, such as FFAR1, GPR119, G protein-coupled bile acid receptor 1 (GPBAR), G protein-coupled receptor 5 (TGR5), calcium-sensing receptor, and GPR142, however, they express GLP-1. In contrast, colonic ECs express various types of GPCR sensors of microbial metabolites, including three receptors for SCFAs, namely olfactory receptor (OLF) 78, OLF558, and FFAR2, and a receptor for aromatic acids, GPR35, a receptor for SBA, GPBAR, and the receptor for acyl-amides and lactate, GPR132. This indicates that nutrient metabolites do not directly stimulate ECs in the small intestine, but through a paracrine mechanism involving GLP-1 secreted from neighboring enteroendocrine cells. Alternatively, colonic ECs are capable of sensing a multitude of different metabolites generated by the gut microbiota and gut hormones, including GLP-1 (Lund et al., 2018).

Moreover, ECs have taste and olfactory receptors that can sense odorous metabolites, including allyl isothiocyanate (Bellono et al., 2017) and isovalerate (Lund et al., 2018), thereby establishing connections with microbiota. Additionally, ECs include abounding endocrine hormone receptors, such as GLP-1, glucose-dependent insulinotropic polypeptide (Lund et al., 2018), and somatostatin (Kidd et al., 2009; Lund et al., 2018), enabling them to regulate glucose homeostasis, lipid metabolism, bone density, and metabolic syndrome-associated diseases, such as obesity and type 2 diabetes (Martin et al., 2017b). Furthermore, ECs express adrenergic receptors, including dopamine, epinephrine, and norepinephrine receptors (Bellono et al., 2017), and CRH receptor 1 (CRH-R1) (Wu et al., 2011), which may enable them to respond to environmental stress (Wong et al., 2019). Notably, ECs have IL-1β receptors and toll-like receptor (TLR)-2,4, indicating that ECs play a role in inflammatory diseases, such as inflammatory bowel disease (IBD) (Kidd et al., 2009). In addition, EC hyperplasia and 5-HT production are immunologically controlled by IL-13 by acting on the IL-13 receptor of ECs (Manocha et al., 2013). IL-13 overexpression in the inflamed mucosa of ulcerative colitis is a unique characteristic of this IBD (Mannon and Reinisch, 2012). Moreover, IL-13 plays a critical role in the pathogenesis of experimental colitis, and ECs-derived 5-HT is an important mediator of IL-13 driven intestinal inflammation (Shajib et al., 2013). This indicates that, in certain cases, ECs serve as pro-inflammatory cells in the gut. More importantly, by suppressing the tumorigenicity 2 receptor (ST2), the unique receptor of IL-33, ECs can detect immune signals for rapid neuroendocrine responses to regulate intestinal homeostasis and host defense against enteric infection (Chen et al., 2020). This suggests that ECs serve as anti-inflammatory cells by sensing immune signals. Owing to the rapid response of ECs to IL-33 via IL-33-ST2 signaling, IL-33 triggers calcium influx for 5-HT release via a PLC-γ1-TRPA1 signaling pathway (Chen et al., 2020). By activating enteric neurons, increased 5-HT enhances intestinal motility and promotes the expulsion of toxins and dead cell bodies from the gut (Bercík et al., 2002; Chen et al., 2020).

ECs can sense the hypoxic environment of the intestines and respond to hypoxia through hypoxia inducible factor 1α (HIF-1α). The GI mucosa is a richly perfused vascular bed directly juxtaposed with an anaerobic and non-sterile lumen in the gut. As such, intestinal epithelial cells, which line the mucosa, experience a uniquely steep physiologic oxygen gradient compared with that of other bodily cells (Taylor and Colgan, 2007). This physiological phenomenon might explain the possibility that ECs become hypoxic sensory cells. Hypoxia induces phosphorylation and activation of TPH1 and promotes 5-HT synthesis (Haugen et al., 2012) via hypoxia transcriptional response element (HRE)-mediated signaling, as HRE is a promoter region for TPH1 (Pocock and Hobert, 2010). Localized hypoxia occurs as a result of chronic inflammation in the gut during pathophysiological processes, including IBD (Taylor and Colgan, 2007). During IBD, increased tissue metabolism and vasculitis render the chronically inflamed mucosa, particularly the epithelium hypoxic, giving rise to the activation of the hypoxia-responsive transcription factor HIF, thus promoting the production of 5-HT (Taylor and Colgan, 2007). Furthermore, ECs express adenosine receptors (Pocock and Hobert, 2010). Hypoxia is associated with increased extracellular adenosine, and increased adenosine hypoxia-induced 5-HT synthesis and secretion is amplified by adenosine receptor 2B signaling and TPH-1 activation (Dammen et al., 2013). Taken together, this indicates that ECs participate in the pathogenesis of intestinal inflammation (Linan-Rico et al., 2016) via HIF-1α and adenosine receptors.

Furthermore, ECs can sense mechanical forces with a mechanosensitive ion channel (Wang et al., 2017; Alcaino et al., 2018). Piezo2 is an important mechano-gated ion channel that is involved in light touch sensitivity and inflammatory allodynia (Yang et al., 2016; Szczot et al., 2018). Recent studies have revealed that mechanical forces in the form of stretching, poking, or stroking of the mucosa stimulated 5-HT release, with the involvement of Piezo2 (Wang et al., 2017; Alcaino et al., 2018). Furthermore, Piezo proteins have been shown to play an important role in mechanical stimulation to induce visceral pain. In patients with IBS, Piezo2 expression in the colon significantly correlates with visceral sensitivity, indicating that Piezo2 is a candidate biomarker for visceral hypersensitivity in IBS (Bai et al., 2017). In rats, Piezo2 knockdown in the dorsal root ganglion (DRG) attenuated visceral sensation to innocuous stimuli in control rats and both innocuous and noxious stimuli with neonatal irritation (Yang et al., 2016). These results suggest a potential mechanism of Piezo2 in visceral hypersensitivity in IBS. More interestingly, a recent study revealed that RNA sensing by ECs Piezo1 is essential for systemic 5-HT synthesis. Piezo1 signaling is a positive regulator of 5-HT production in ECs. However, Piezo1 is dispensable for mechanical force-induced 5-HT production in the gut (Sugisawa et al., 2020). The significant findings regarding the important role of Piezo2 and Piezo1 might provide important perspectives to investigate novel mechanisms of ECs-related diseases, such as IBS.

Notably, ECs also expressed 5-HTR. Studies have indicated that stimulatory 5-HT_1A_R, 5-HT_2_R, 5-HT_3_R, and inhibitory 5-HT_4_R are present on ECs of porcine and human intestinal mucosa (Schwörer and Ramadori, 1998). This suggests that these contradictory roles of 5-HTR in ECs may regulate 5-HT production via an autocrine mechanism.

## Enterochromaffin Cells-Derived 5-Hydroxytryptamine Synthesis, Secretion, and Metabolism

**FIGURE 2:**
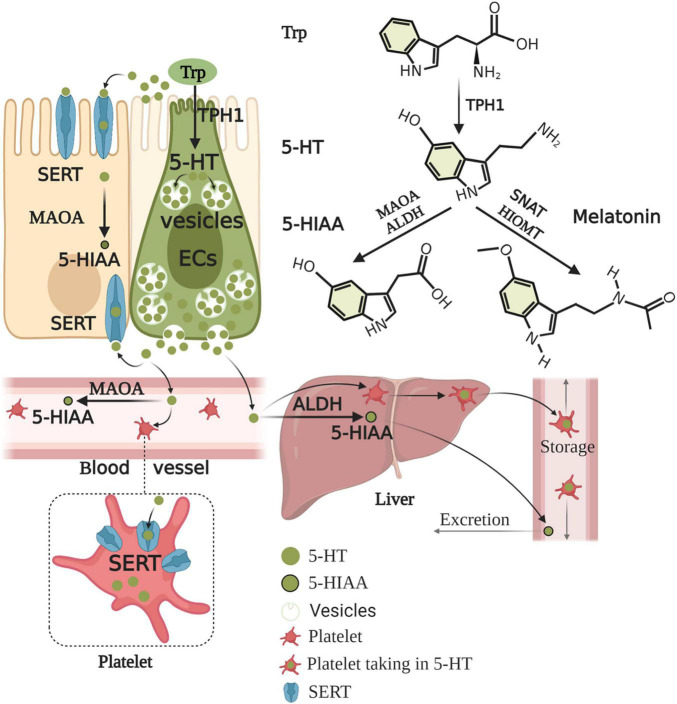
Enterochromaffin cells synthesis of serotonin and its metabolism and excretion. Trp is converted to 5-HTP by TPH1 of ECs. 5-HTP is then metabolized to 5-HT by AADC. Upon production, 5-HT is stored in large dense core vesicles of ECs and prepared for release when required by the body. 5-HT acts on objective cells to exert nutrition, intestinal motility, secretion, and proinflammatory and anti-inflammatory effects. 5-HT is released into the lumen and lamina propria and can enter the blood in the lamina propria. 5-HT must be rapidly removed to prevent excessive activation and/or receptor desensitization. 5-HT is taken up by almost all epithelial cells, nerves, immune cells, and platelets via SERT. 5-HT is inactive and converted to 5-HIAA by enterocytes and enteric neurons with MAOA. The portal circulation is first processed by the liver before the blood enters the systemic circulation. Free 5-HT in the blood is rapidly degraded by MAOA or ALDH in the liver, while 5-HT in platelets is protected from degradation. Thus, generally, only 5-HT stored in platelets enters the general circulation. 5-HIAA in the general circulation is finally excreted through the urine. Conversely, 5-HT can also be further metabolized to melatonin by SNAT and HIOMT. Trp, tryptophan; 5-HTP, 5-hydroxy-L-tryptophan; TPH1, tryptophan hydroxylase 1; AADC, aromatic amino acid decarboxylase; 5-HT, serotonin; SERT, serotonin reuptake transporter; MAOA, monoamine oxidase A; 5-HIAA, 5-hydroxyindole acetic acid; ALDH, aldehyde dehydrogenase; SNAT, serotonin N-acetyltransferase; HIOMT, hydroxyindole O-methyltransferase.

ECs account for only 1% of enteric epithelial cells but secrete 90% of 5-HT in the body (Mawe and Hoffman, 2013). Considering 5-HT content in the gut alone, approximately 95% is synthesized in ECs, whereas 5% is synthesized in enteric neurons and other cell types (Banskota et al., 2019). ECs produce 5-HT from its precursor, L-tryptophan (Trp). The first rate-limiting step of 5-HT biosynthesis is the transformation of L-Trp into 5-hydroxy-L-tryptophan (5-HTP) catalyzed by TPH. The second step is the decarboxylation of 5-HTP by L-amino acid decarboxylase, leading to 5-HT production (Banskota et al., 2019). The rate-limiting enzyme for 5-HT biosynthesis is TPH, which includes TPH1 in ECs and TPH2 in the ENS and CNS (Walther et al., 2003). 5-HT, as a paracrine modulator produced, stored, and released from ECs, is delivered to neighboring cells through 5-HTR (Wood, 2020). Upon release by ECs, 5-HT may take several possible routes. First, 5-HT is released into the lumen or taken up by epithelial cells via the 5-HT reuptake transporter (SERT) (Bertrand and Bertrand, 2010). 5-HT released into the lamina propria, where 5-HT may act with 5-HTR in epithelial cells, enteric neurons, and mast cells (MCs), to exert intestinal motility, secretion, proinflammatory, and anti-inflammatory effects (Martel et al., 2003), may also be taken up into the enterocytes by SERT or enter the blood (Bertrand and Bertrand, 2010). 5-HT is present as free 5-HT in the blood or taken up by platelets via SERT. The portal circulation is first processed by the liver before the blood enters the systemic circulation. Free 5-HT in the blood is rapidly degraded by monoamine oxidase A (MAOA) to 5-HIAA or by glucuronidases in the liver, while 5-HT in platelets is protected from degradation. Therefore, only 5-HT stored in platelets enters the general circulation (Bertrand and Bertrand, 2010). After 5-HT acts on one of its receptors, 5-HT must be removed rapidly to prevent excessive activation and/or receptor desensitization. 5-HT inactivation requires transmembrane transport because 5-HT cannot be catabolized extracellularly, and 5-HT is charged at a physiological pH and, therefore, scarcely traverses plasma membrane lipid bilayers (Gershon and Tack, 2007). Thus, the inactivation of 5-HT requires the mediation of a selective plasmalemma, sodium-dependent SERT (Blakely et al., 1994). Epithelial cells and enteric serotonergic neurons express SERT (Wade et al., 1996), which take up and inactivate 5-HT (Bertrand and Bertrand, 2010). All of the epithelial cells in the intestinal lining appear to express SERT, therefore, these transporters act as selective sponges to remove 5-HT from the interstitial space after release by ECs. Therefore, SERT serves as a critical molecule in the local regulation of 5-HT availability and action in the intestines (Mawe and Hoffman, 2013). Studies on the intestine during early postnatal development and adulthood revealed that decreased SERT function, via reduced expression levels or pharmacological blockade (Bian et al., 2007), leads to high extracellular 5-HT levels. SERT inhibition or deletion reinforces 5-HT-mediated responses, while increasing SERT activity diminishes responses to 5-HT (Margolis et al., 2016). Alternatively, 5-HT in the lamina propria can be taken up by SERT-expressing cells, such as T cells and B cells, which also activate the immune response (Spohn and Mawe, 2017). Consequently, the effects of 5-HT in the gut are balanced by 5-HT secretion, catabolism, and uptake mechanisms. In contrast to neurons, ECs-5-HT is conceivably stored in large dense core vesicles in complex with large acidic chromogranin proteins (Machado et al., 2010). ECs synthesis of serotonin and its metabolism and excretion are summarized in Figure 2.

## Functions of Enterochromaffin Cells-Derived 5-Hydroxytryptamine in the Gut

5-HT, which plays a key role in regulating intestinal motility, intestinal permeability, and visceral sensation, and participates in emotional regulation, such as anxiety and depression (De Deurwaerdère et al., 2020), is an important neurotransmitter in the CNS and ENS. 5-HT activates 5-HTR to exert its biological functions. There are seven types of 5-HTR (5-HT_1–7_R), and at least 14 subtypes are widely distributed in the intestine and brain (De Deurwaerdère et al., 2020).

The GI wall consists of mucous, submucous, muscular, and serosal layers (Matsumura et al., 2020). Epithelial cells, M-cells, ECs, goblet cells, and other enteroendocrine cells are located in the mucous layer. Inflammatory cells, MCs, blood vessels, and afferent nerve terminals are located in the submucous, namely the lamina propria. These cells and neurons express various 5-HTR (Wood, 2020). 5-HT released from ECs mediates numerous GI functions, including peristalsis, secretion, vasodilation, and perception of pain or nausea, through activation of a diverse family of 5-HTR on intrinsic and extrinsic afferent nerve fibers located in the lamina propria (Mawe and Hoffman, 2013). Mucosal ECs release 5-HT as a paracrine signaling molecule that acts on neighboring cells and neurons (Pan and Gershon, 2000). For example, 5-HT acting on 5-HT_4_R stimulates epithelial cells to secrete electrolytes and H_2_O into the intestinal lumen and stimulates goblet cells to secrete mucus (Mawe and Hoffman, 2013; Wood, 2020). ECs have been shown to directly interact with afferent vagal GPR65-neurones expressing 5HT_3_R (Bellono et al., 2017). Morphological studies have demonstrated that myenteric Dogiel type 2 neurons project into the mucosa with nerve endings in close proximity to ECs (Neunlist et al., 1999). Additionally, 5-HT spreads under the epithelial layer and enters the lamina propria. 5-HT acting on 5-HT_1A_R induces MC adhesion, migration, and degranulation (Wang et al., 2013; Wood, 2020). Released mediators from MCs become paracrine signals to the ENS, spinal afferents, and secretory glands (Wang et al., 2013). Clinical studies have shown that, compared with healthy controls, patients with IBS-D exhibited a significant increase in 5-HT release and correlated with MC counts. A significant correlation was found between mucosal 5-HT release and the severity of abdominal pain in patients (Cremon et al., 2011). 5-HT released from ECs stimulates intestinal motor and secretomotor reflexes by activating 5-HTR on terminals of intrinsic primary afferent neurons (IPANS), the cell bodies of which are located in the enteric nerve plexuses. IPANS synapse with interneurons and activate other enteric neurons to initiate a reflex response. ECs-derived 5-HT was also applied at 5-HTR on extrinsic sensory nerve terminals to convey nociceptive signals to the CNS (via spinal afferents) or to initiate GI reflexes via vagal afferents (Galligan, 2017). In addition, 5-HT acting on 5-HT_3_R of spinal afferent nerve terminals (Kozlowski et al., 2000) or ENS is involved in processes associated with emotion, cognition, memory, pain perception, and GI functions, including secretion and motility (Kato, 2013). When acting on 5-HT_3_R of vagal afferent nerve terminals, 5-HT engages in several physiological and pathophysiological conditions (Glatzle et al., 2002), including distention- and chemical-evoked vagal reflexes, nausea, vomiting, and visceral hypersensitivity (Browning, 2015). 5-HT acting on 5-HT_7_R in immune cells, such as monocytes, lymphocytes, and dendritic cells, may play a crucial role in inflammation signaling (Quintero-Villegas and Valdés-Ferrer, 2019; Wu et al., 2019). Furthermore, 5-HT acting on 5-HT_1A_R in monocytes and dendritic cells has anti-inflammatory effects (Hernández-Torres et al., 2018).

**FIGURE 3:**
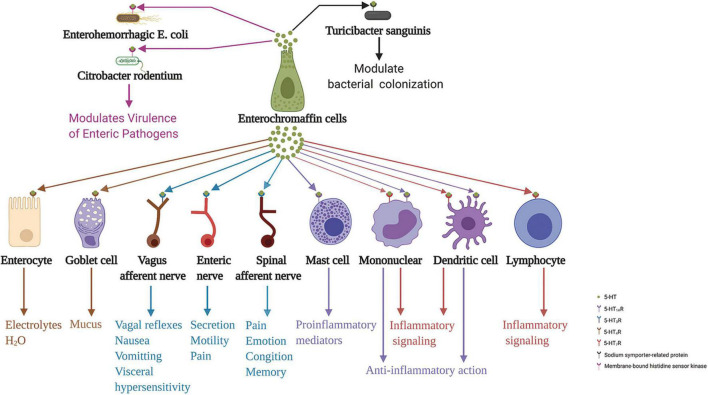
Enterochromaffin cells action on various neighbor cells, neurons, and intestinal microbiota through released 5-HT. 5-HT acting on 5-HT_4_R stimulates epithelial cells to secrete electrolytes and H_2_O into the intestinal lumen and stimulates goblet cells to secrete mucus. Moreover, 5-HT acting on 5-HT_3_R of terminals of the spinal and vagus afferent nerves, and enteric nerves are involved in processes associated with emotion, cognition, memory, pain perception, distention- and chemical-evoked vagal reflexes, nausea, vomiting, visceral hypersensitivity, and intestinal functions, including secretion and motility. Furthermore, 5-HT acting on 5-HT_1A_R induces mast cell adhesion, migration, and degranulation. In addition, 5-HT acting on 5-HT_7_R in immune cells, including monocytes, lymphocytes, and dendritic cells, may be implicated in inflammation signaling. In contrast, 5-HT acting on 5-HT_1A_R in monocytes and dendritic cells has an anti-inflammatory role. In addition to these functions, 5-HT action on 5-HT receptors of enteric bacteria, sodium symporter-related protein of *Turicibacter sanguinis*, and membrane-bound histidine sensor kinase of enterohemorrhagic *Escherichia coli* and *Citrobacter rodentium*, play a crucial role in modulating bacterial colonization and virulence of enteric pathogens. 5-HT, serotonin; 5-HTR, serotonin receptor.

ECs secrete 5-HT basally and act on adjacent exciting cells and enteric neurons. Conversely, it also secretes 5-HT apically to the gut lumen (Fujimiya et al., 1997). The gut microbiota regulates 5-HT levels in the intestinal epithelium and lumen (Reigstad et al., 2015). However, whether 5-HT in the intestinal lumen acts on the intestinal microbiota has been unclear until recently. A recent study demonstrated that elevated intestinal luminal 5-HT levels by oral supplementation or genetic deficiency of SERT in the host increases the relative abundance of spore-forming members of the gut microbiota. The study identified *Turicibacter sanguinis* as a gut bacterium that expresses a neurotransmitter sodium symporter-related protein with sequence and structural homology to mammalian SERT (Fung et al., 2019). Coincidentally, Kumar et al. (2020) indicated that 5-HT secreted into the lumen can decrease virulence gene expression in enterohemorrhagic *Escherichia coli* and *Citrobacter rodentium*. They stated that the membrane-bound histidine sensor kinase CpxA is a bacterial 5-HT receptor. 5-HT induces dephosphorylation of CpxA, which inactivates the transcription factor CpxR and controls the expression of virulence genes. Therefore, repurposing 5-HT agonists to inhibit CpxA may represent a potential therapeutic intervention for enteric bacteria. These results suggest the vital role of ECs in the communication between the gut and enteric microbiota. ECs action on various neighbor cells, neurons, and intestinal microbiota are summarized in Figure 3.

## Factors Affecting Enterochromaffin Cells Synthesis of 5-Hydroxytryptamine

ECs regulation and 5-HT release have been studied since their discovery in the 1950s, however, progress has been slow due to methodological problems in diligently measuring 5-HT release and the fact that ECs, like other enteroendocrine cell populations, are a minor population of cells scattered and outnumbered by other cell types (Lund et al., 2018). Moreover, it was found that a large proportion of ECs renewed relatively rapidly, with a turnover rate of approximately 16 days in the gut (de Bruïne et al., 1992). Although these disadvantages severely impede the in-depth study of ECs physiology (Wang et al., 2020), in addition to the selection of human pancreatic neuroendocrine tumor cell line (BON) cells (Christofi et al., 2004; Wu et al., 2011) and the malignant ECs carcinoid cell line (Siddique et al., 2009), the ECs model has performed well, despite the fact that tumor cell lines have not proven to be suitable mimics of intestinal ECs physiological responses (Wang et al., 2020). In addition, a study revealed that a human pancreatic endocrine cell line, one islet carcinoma cell line (QGP-1), also expressed various ECs marker genes, including TRPA1 and TPH1, suggesting that QGP-1 is a new model for the investigation of ECs (Doihara et al., 2009). Therefore, multiple subsequent studies used this model to conduct ECs-related research (Kalbe et al., 2016; Herrera-Martínez et al., 2020). In addition, the milestone emergence of intestinal organoids (Sato et al., 2009; Buske et al., 2012) enables a broad *in vitro* study of ECs from cell to tissue levels (Tsuruta et al., 2016). Currently, the technology of single-cell sequencing has moved ECs-related research into a new era (Fischer et al., 2019; Gehart et al., 2019).

**FIGURE 4:**
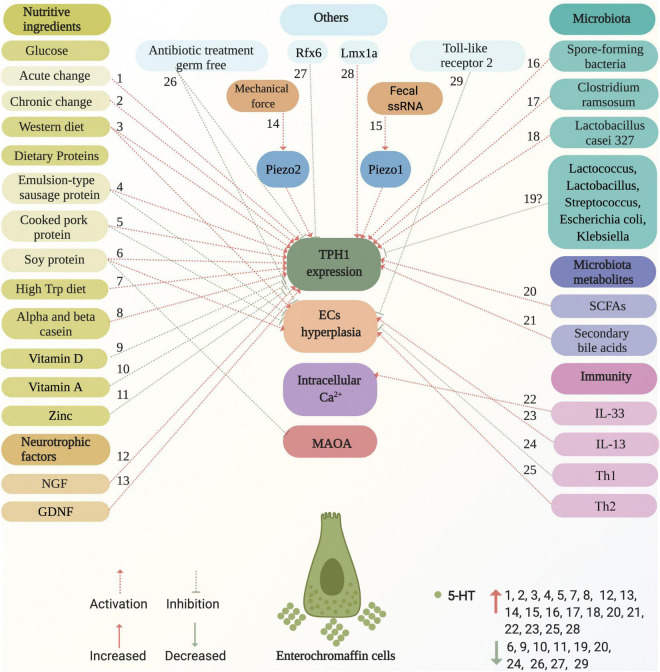
Factors and potential mechanisms of regulating enterochromaffin cells synthesis of 5-HT. Various factors activate or inhibit ECs synthesis of 5-HT, including nutritive ingredients (glucose, dietary proteins, western diet, high Trp diet, casein, vitamin D, vitamin A, and zinc), neurotrophic factors (NGF and GDNF), microbiota (spore-forming bacteria and *Clostridium ramosum*), metabolites (SCFAs, such as acetate, propionate, butyrate, and secondary bile acids), immunity (IL-33, IL-13, TH1, and TH2), mechanical force, fecal ssRNA, as well as others (germ-free, Rfx6, Lmx1a, and antibiotic treatment). These are marked with red and green dotted arrows for activation and inhibition, respectively. The potential mechanisms of factors that act on ECs are involved in the effects on TPH1 expression, ECs hyperplasia, intracellular Ca^2+^, and MAOA. Factors that increase and decrease 5-HT levels are marked with red up and green down arrows, respectively. ECs, enterochromaffin cells; NGF, neuron growth factor; GDNF, glial cell-derived neurotrophic factor; ssRNA, single-stranded RNA; TPH1, tryptophan hydroxylase 1; MAOA, monoamine oxidase A; SCFAs, short chain fatty acids; Trp, tryptophan; Th, T helper.

ECs are located at the forefront of the gut and link with the adjacent intestinal epithelial cells, enteric neurons, vagus nerve afferent terminals, and spinal nerve afferent terminals, and express numerous functional receptors. In addition, TPH1 is a rate-limiting enzyme in the synthesis of 5-HT. Accordingly, intraluminal-, intestinal-, enteric neuron-, vagus nerve afferent terminal-, and spinal nerve afferent terminal-derived stimuli may directly or indirectly promote or inhibit ECs to produce 5-HT by increasing or decreasing the number of ECs and upregulating or downregulating TPH1 expression. In contrast, novel ion channels were found to be important for ECs excitability and 5-HT release (Bellono et al., 2017; Strege et al., 2017). Mechanistically, the combination of ligands and receptors on ECs caused Ca^2+^ influx, elevation of intracellular free Ca^2+^ levels, and, consequently, 5-HT release (Braun et al., 2007). The factors and potential mechanisms that regulate the ECs synthesis of 5-HT are summarized in Figure 4.

Glucose activates the secretion of various cell types and is the leading absorbed form of carbohydrates in the gut. Zelkas et al. (2015) reported that primary ECs respond to acute changes in glucose availability through increases in intracellular Ca^2+^ activation of 5-HT secretion, but respond to chronic changes in glucose levels through the upregulation of TPH1 expression. A previous study in mice with oral polysaccharide administration that increased carbohydrate availability promoted intestinal 5-HT synthesis (Kashyap et al., 2013), however, the underlying mechanism was not elucidated.

In rats, a western diet (high-fat diet; HFD) significantly increased ECs and TPH1 mRNA and decreased SERT mRNA levels and protein expression in the small intestine (Bertrand et al., 2011; Crane et al., 2015). Furthermore, mice fed a HFD showed increased body weight, hyperglycemia, and impaired glucose tolerance, and generated anxiogenic-like/depressive-like symptoms. This phenotype is associated with decreased extracellular 5-HT levels in the hippocampus (Zemdegs et al., 2016). However, the precise mechanism of action is unclear. It is well known that two main pathways of 5-HT synthesis, the gut and the brain, are separate (Bader, 2020). A HFD increases the number of ECs and the TPH1 mRNA level, thus implying elevation of 5-HT secretion in the gut, which might mean a reduction in the synthesis of 5-HT in the brain in the case of the same total amount of Trp entering the gut. The traditional view that 5-HT synthesis in the gut and brain is separate has been challenged (Zemdegs et al., 2016) despite the lack of more convincing evidence. Existing evidence indicates that the regulation of ECs producing 5-HT might affect 5-HT generation in the brain. Similarly, Porter et al. (2008) revealed that TPH1 is associated with the regulation of peripheral Trp levels and, therefore, the availability of Trp to the brain. Evidence from studies on germ-free (GF) and antibiotic-treated mice also supports this view. In the body, serum, plasma, colonic, and fecal concentrations of 5-HT are substantially reduced compared to conventionally raised controls (Uribe et al., 1994; Wikoff et al., 2009; Sjögren et al., 2012; Yano et al., 2015), and are associated with decreases in TPH1 expression and ECs density, particularly in the colon (Uribe et al., 1994; Yano et al., 2015). In parallel, GF mice had reduced anxiety compared to specific pathogen-free (SPF) mice with a normal gut microbiota (Diaz Heijtz et al., 2011; Lukić et al., 2019; Pan et al., 2019), and a significant elevation in the concentration of 5-HT and 5-hydroxyindoleacetic acid in the medial prefrontal cortex and hippocampus accompanied by increased concentrations of Trp in the plasma (Clarke et al., 2013; Lukić et al., 2019). However, no increased TPH2 expression was detected, which was responsible for the synthesis of 5-HT from Trp in the brain, suggesting the possibility of altered intrinsic activity of this enzyme in GF animals (Clarke et al., 2013). However, Lukić et al. (2019) suggested higher TPH2 expression in the dorsal raphe nucleus of GF mice than in that of SPF mice.

Dietary proteins can also regulate 5-HT biosynthesis. Xie et al. (2020) showed that emulsion-type sausage protein and cooked pork protein diets increase TPH1 mRNA expression and 5-HT levels, but reduce the number of ECs. In contrast, a soy protein diet increases the number of ECs and TPH1 mRNA levels, but decreases the MAOA mRNA level and 5-HT content. Casein, particularly alpha and beta casein proteins, is beneficial in stimulating enteroendocrine cell lines, STC-1 cells, proliferation, and GLP-1 secretion (Gillespie and Green, 2016). STC-1 cells also secrete 5-HT (Ripken et al., 2016). In addition, caseinate hydrolysate, lead functional compound-25 (LFC25), significantly increases calcium signaling in STC-1 (O’Halloran et al., 2018), demonstrating the promotion of 5-HT secretion by LFC25. In addition, beta-lactoglobulin and alpha-lactalbumin may also play functional roles (Gillespie et al., 2015).

Trp, the precursor of 5-HT, is an essential amino acid for animals and humans and is mainly derived from diet (Gao et al., 2020). After entering the gut, 5-HT synthesis and kynurenine degradation are the main pathways for Trp metabolism (Gao et al., 2020). In pregnant rats fed a high-Trp diet, hyperserotonemia was detected in experimental pups compared to controls, suggesting increased 5-HT production in ECs (Musumeci et al., 2014). However, formula feeding reduced the number of ECs and 5-HT concentration, but increased tryptamine levels relative to sow feeding, indicating that the formula diet driving microbiota shifted Trp metabolism from 5-HT to tryptamine in the neonatal porcine colon (Saraf et al., 2017).

In addition to the nutritional components that affect ECs release of 5-HT, certain specific microbiota also promote EC secretion of 5-HT. Yano et al. (2015) revealed that indigenous spore-forming bacteria from the mouse and human microbiota promote 5-HT biosynthesis from colonic ECs with increased TPH1 expression. This suggests that microbiota primarily modulate 5-HT metabolism by affecting the host colonic ECs. Mandić et al. (2019) reported that *Clostridium ramosum* regulates ECs development and 5-HT release, including upregulation of TPH1 expression. Additionally, certain probiotic *Lactobacillus* strains, such as *Lactobacillus casei 327*, can indirectly promote colonic 5-HT synthesis by increasing TPH1 expression (Hara et al., 2018). Interestingly, certain commensal microbiota can directly utilize luminal Trp for 5-HT synthesis, such as *Lactococcus*, *Lactobacillus*, *Streptococcus*, *E. coli*, and *Klebsiella*, which produce 5-HT by expressing TPH (O’Mahony et al., 2015). These microbiota may indirectly inhibit ECs to synthesize 5-HT (Saraf et al., 2017). Furthermore, intestinal flora metabolites potently influenced the ECs synthesis of 5-HT. Accumulating evidence has shown that SCFAs promote 5-HT production (Fukumoto et al., 2003; Akiba et al., 2015; Reigstad et al., 2015) by acting on SCFA receptors of ECs (Nøhr et al., 2013; Martin et al., 2017a). Moreover, SCFAs are the major products of microbial carbohydrate metabolism and can suppress kynurenine production from Trp (Kennedy et al., 2017; Gao et al., 2018), and therefore, indirectly promote 5-HT production from Trp (Gao et al., 2020). In addition, adding indigestible carbohydrates, such as fructo-oligosaccharide and resistant starch, increases carbohydrate availability and leads to increased SCFA production (Zhou et al., 2017), thus enhancing 5-HT synthesis.

Importantly, intestinal homeostasis requires a tightly regulated balance between ISC proliferation and differentiation (Santos et al., 2018). ECs are differentiated by ISC via a specific signaling pathway (Schonhoff et al., 2004). Thus, alteration of this pathway may lead to changes in ECs. A recent study showed that ELS triggers nerve growth factor (NGF) elevation, which directly targets ISC, stimulating their expansion and differentiation by trans-activating Wnt/β-catenin signaling, thus leading to intestinal ECs hyperplasia and 5-HT increase (Wong et al., 2019). Furthermore, Lin et al. (2020) showed that glial cell-derived neurotrophic factor (GDNF) rearranged during transfection is crucial for regulating ISC and ECs differentiation in wrap-restraint stress mice and *in vitro*. GDNF treatment amplifies Wnt signaling and increases 5-HT levels in colonic organoids in a dose-dependent manner. This robust evidence suggests that NGF and GDNF affect ISC differentiation toward the Wnt signal, inducing ECs hyperplasia; thus, inhibition of this process might help to restore intestinal homeostasis (Koch et al., 2011; Metidji et al., 2018). Additionally, a recent study using single cell and bulk RNA sequences showed that enteroendocrine progenitors differentiated into two main cell trajectories, ECs and peptidergic enteroendocrine cells, the differentiation programs of which are differentially regulated by regulatory factor X-box binding transcriptional factor 6 (Rfx6) transcription. Rfx6 represses LIM homeobox transcription factor 1 alpha (Lmx1a) and TPH1, two genes essential for 5-HT biosynthesis. In the absence of intestinal Rfx6, the number of 5-HT-producing ECs and mucosal 5-HT content increases (Piccand et al., 2019). Indeed, Gross et al. (2016) showed that the transcription factor Lmx1a is expressed in ECs and is considered a novel ECs marker, which is also essential for the production of the 5-HT biosynthetic enzyme TPH1.

In addition, the immune system is related to the ECs-5-HT signal, indicating that the neuroendocrine axis plays a role in this process (Chen et al., 2020). As mentioned above, Chen et al. (2020) revealed that IL-33-ST2 signaling selectively promoted ECs-derived 5-HT secretion. IL-33 triggers calcium influx for 5-HT release via the PLC-γ1-TRPA1 signaling pathway. Their study highlighted the importance of establishing an immune-neuroendocrine axis in calibrating rapid 5-HT release for intestinal homeostasis. In addition, as mentioned above, BON cells express the IL-13 receptor and produce more 5-HT in response to IL-13 (Manocha et al., 2013). In dextran sulfate sodium (DSS)-induced colitis in a mouse model, IL-13^–/–^ mice administered DSS exhibited significantly reduced colitis severity compared to that of wild-type (WT) mice, accompanied by downregulation of ECs number and colonic 5-HT content. These results demonstrate that IL-13 plays a critical role in the pathogenesis of experimental colitis, and 5-HT is an important mediator of IL-13-driven intestinal inflammation (Shajib et al., 2013). In another study, both severe combined immunodeficient (SCID) mice and WT controls were infected with the nematode *Trichuris muris*. After infection, the number of ECs and the amount of 5-HT were significantly lower in SCID mice than in WT mice. The number of ECs and the level of 5-HT were significantly increased after reconstructing SCID mice with CD4^+^ T cells from infected mice, accompanied by an upregulation of colonic CD3^+^ T cells and T helper 2 (Th2) cytokines. These results showed an important immunoendocrine axis in the gut, where secretory products from CD4^+^ T cells interact with ECs to heighten 5-HT production in the gut via Th2-based mechanisms. Moreover, increased ECs number and 5-HT content were found in mice treated with *in vitro* polarized Th2 cells or in mice with impaired T helper (Th) 1 cytokine production, indicating that ECs number is closely correlated with intestinal Th1/Th2 balance (Motomura et al., 2008). *In vitro* studies also revealed that the key cytokines of the Th1 response, such as interferon-γ and interferon-β, significantly inhibit the proliferation of ECs models, including BON and QGP-1 cells (Detjen et al., 2002; Höpfner et al., 2004; Vitale et al., 2006). This evidence indicates that the number of ECs might be influenced by Th1 or Th2 cytokine-predominant environments. It appears that Th2-related cytokines might contribute, and Th1-related cytokines may inhibit, the development of ECs hyperplasia in the gut. Moreover, a recent study indicated that TLR-2 plays a vital role in mediating mucosal 5-HT production in the gut. Antibiotic treatment reduced the number of ECs and 5-HT levels in naive C57BL/6 mice, which was associated with TLR-2 downregulation. TLR-2-deficient mice express lower numbers of ECs and 5-HT levels. Besides, excretory-secretory products from *T. muris* induce increased 5-HT production in BON-1 cells via TLR-2 in a dose-dependent manner (Wang et al., 2019). The results provided novel insights into the potential benefits of targeting TLR-2 in various gut disorders that exhibit aberrant 5-HT signaling, such as IBS. Together, manipulating specific immune systems can regulate ECs function.

Mechanically, the ion channels of ECs are decisive factors for the release of 5-HT. Strege et al. (2017) demonstrated that Na(V)1.3 is critical for generating action potentials in ECs, and is also important for regulating 5-HT release by these cells. Na(v)1.3 knockdown in the lumbar 4 DRG results in the attenuation of nerve injury-induced mechanical allodynia in a spared nerve injury animal model (Samad et al., 2013). Besides, single ECs function analysis revealed that Ca^2+^ enters ECs upon stimulation and triggers quantal 5-HT release via L-type Ca^2+^ channels. Local 5-HT levels are likely to be maintained around the activation threshold for mucosal 5-HT receptors, which is dependent upon stimulation and location within the GI tract (Raghupathi et al., 2013). Moreover, in BON or ECs isolated from human gut surgical specimens, uridine-5′-triphosphate activates a predominant P2Y4R pathway to trigger Ca^2+^ oscillations via internal Ca^2+^ mobilization through a PLC/IP3/IP3R/SERCA Ca^2+^ (voltage-sensitive Ca^2+^ currents, ICa) signaling pathway to stimulate 5-HT release (Liñán-Rico et al., 2017). While chemical ion channels, such as Na(v)1.3, L-type Ca^2+^, and ICa, play a crucial role in 5-HT release under different conditions, the mechanical ion channel plays a role in transferring mechanical force to 5-HT release (Chin et al., 2012; Wang et al., 2017; Alcaino et al., 2018). Piezo2 is an important mechano-gated ion channel involved in light touch sensitivity and inflammatory allodynia (Yang et al., 2016; Szczot et al., 2018). In recent years, mechanical stimulation using a rhythmic flex model induced transcription and activation of TPH1 and vesicular monoamine transporter 1 and the release of 5-HT in IBD human ECs and neoplastic ECs (Chin et al., 2012). However, the underlying molecular mechanism is unclear. Recently, Wang et al. (2017) first reported that the mechanosensitive ion channel Piezo2 was specifically expressed in human and murine small bowel ECs. Activation of Piezo2 by mechanical forces results in a characteristic ionic current, the release of 5-HT, and stimulation of GI secretion. Piezo2 is critical for ECs mechanotransduction. Later, Alcaino et al. (2018) suggested that Piezo2 was expressed in a subset of murine enteroendocrine cells and ECs, and it was distributed near 5-HT vesicles by super-resolution microscopy. Mechanical stimulation induces a Piezo2-dependent increase in intracellular Ca^2+^ and 5-HT release. Whereas, conditional knockout of intestinal epithelial Piezo2 results in a significant decrease in mechanically stimulated epithelial secretion. Furthermore, Bai et al. (2017) used post-infectious IBS mice to show that Piezo2 was more abundant in the colon than in the small intestine. Piezo2 expression in the colon is significantly correlated with visceral sensitivity rather than mucosal inflammation, indicating that Piezo2 is a candidate biomarker for visceral hypersensitivity in IBS. Piezo2 knockdown in the DRG attenuates visceral sensation to innocuous stimuli in control rats and both innocuous and noxious stimuli in rats with neonatal irritation (Yang et al., 2016). As with Piezo2, Piezo1 plays a pivotal role in 5-HT release. Recently, the study of Piezo1 with ECs has attracted considerable attention. Sugisawa et al. (2020) revealed the molecular mechanisms by which intestinal microbiota control 5-HT production. They identified that ssRNA is a natural Piezo1 ligand, where stimulation of Piezo1 with ssRNA induces Piezo1-dependent calcium flux, TPH1 upregulation, and, thus, elevation of serum 5-HT levels. Mice that lacked Piezo1 in the intestinal epithelium reduced 50% serum and intestinal levels of 5-HT due to decreased TPH1 expression, without changes in the quantities of ECs. However, mechanical force-induced Piezo2 activation occurs in a Piezo1-dispensable manner (Matute et al., 2020; Sugisawa et al., 2020). This study was the first to show that a classic mechanotransducer could also act as an ssRNA extracellular receptor (Matute et al., 2020). This profound result, a novel ssRNA-Piezo1-TPH1-5-HT axis, may provide new insights into disorders of microbiota–gut–brain interactions.

In addition, certain micronutrients also affect ECs function. Oral zinc supplementation is considered an effective treatment for acute diarrhea in children (Dalfa et al., 2018), and the WHO recommends oral zinc supplementation for rotavirus (RV)-induced diarrhea management (Gandhi et al., 2016). RV induces ECs production of 5-HT (Hagbom et al., 2011), thus zinc might act on ECs. Indeed, *in vitro* piglet small intestinal epithelium, serosal zinc attenuates 5-HT and vasoactive intestinal peptide (VIP)-induced secretion (Carlson et al., 2008). Additionally, a recent study revealed that ZnT8, a zinc transporter, is expressed in enteroendocrine cells, particularly in 5-HT positive ECs. The lack of ZnT8 results in an elevated circulating 5-HT level owing to enhanced TPH1 expression (Mao et al., 2019). This suggests that ZnT8 expression in ECs plays a role in the balance of TPH1 expression, which might be due to the inhibition effect. Oral zinc supplementation might bind ZnT8 in ECs and act to downregulate TPH1. However, the evidence remains insufficient, and further studies are required. Moreover, Guo et al. (2018) demonstrated that vitamin A supplementation significantly reduces the mRNA expression of TPH1 and 5-HT in children with autism. Retinoic acid (RA) is the main derivative of vitamin A. RA targets the RA receptor (RAR) to mediate signal transduction. RAR has three isoforms: RAR α, β, and γ. It was confirmed using Genomatix^1^ that TPH1 contained a putative binding site for RARγ in the promoter region of TPH1, and vitamin A was considered to regulate the mRNA level of TPH1 via RARs. In addition, vitamin D regulates 5-HT synthesis. A large *in silico* and microarray-based study previously identified putative DR3 vitamin D response elements (VDREs) upstream of the TPH1 promoter regions (Wang et al., 2005). Vitamin D acts on the VDRE and inhibits the transcription of TPH1 in tissues outside the blood–brain barrier, thus decreasing the production of 5-HT in ECs (Patrick and Ames, 2014).

## Evidence for Early Life Stress-Induced Irritable Bowel Syndrome

**FIGURE 5:**
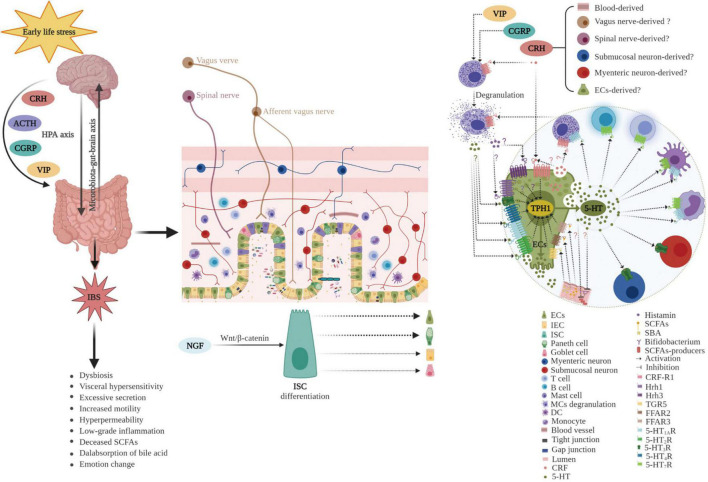
Potential role of enterochromaffin cells in early life stress-induced irritable bowel syndrome. ELS chronically activates the HPA axis and increases the levels of neuropeptides, such as CRH, CGRP, VIP, and ACTH. Furthermore, ELS disrupts intestinal homeostasis and the microbiota–gut–brain axis through various pathways including neuroendocrine and immune activation (such as degranulation of mast cells), differentiation of ISC toward secretory lineages including ECs, alterations in the composition of intestinal microbiota (decreased abundance of SCFAs producers, such as *Bifidobacterium*), and thus, changes in metabolites (including reduced SCFAs and increased SBA). Moreover, hyperplasia of ECs and upregulation of TPH1 result in excessive 5-HT release. 5-HT acts on various 5-HT receptors in neighboring cells and nerves, inducing visceral hypersensitivity, excessive secretion, increased motility, hyperpermeability, and the genesis of anxiety or depression. ECs play a vital role in the pathogenesis of ELS-induced IBS. ECs, enterochromaffin cells; ELS, early life stress; HPA, hypothalamic–pituitary–adrenal; CRH, corticotropin releasing hormone; CGRP, calcitonin gene-related peptide; VIP, vasoactive intestinal peptide; ACTH, adrenocorticotropic hormone; ISC, intestinal stem cell; IEC, intestinal epithelial cell; SCFAs, short chain fatty acids; SBA, secondary bile acids; TPH1, tryptophan hydroxylase 1; 5-HT, serotonin; MCs, mast cells; DC, dendritic cell; Hrh, histamine H receptor; TGR5, G protein-coupled receptor 5; FFAR, free fatty acid receptor; NGF, neurotrophic factors.

Early childhood is a critical developmental period, and ELS may increase the predisposition to GI diseases, including IBS, in later life (Wong et al., 2019; Jones et al., 2020; Ju et al., 2020; Low et al., 2020). Bradford et al. (2012) investigated different early adverse life events and their association with IBS, and demonstrated that IBS patients had higher incidences of general trauma, physical punishment, and emotional abuse compared with controls. Moreover, emotional abuse was identified as the strongest predictive factor of IBS. Furthermore, ELS is correlated with the severity of IBS symptoms (Park et al., 2016). In addition, a recent study suggested that resilience, the ability to adapt positively to stress and adversity, was lower in IBS patients (*n* = 820) than in the general population (*n* = 1026; *P* < 0.001) and was associated with more severe IBS symptoms. ELS decreases the ability to overcome adversity in both IBS patients and the general population (Parker et al., 2020). Together, these results provide evidence for the potent role of ELS in the pathogenesis of IBS. However, the molecular mechanisms remain to be elucidated. Dysbiosis in the commensal bacterial communities, increased intestinal permeability, irregular intestinal motility, visceral hypersensitivity, and anxiety/depression-like behaviors have been observed in animal models (Labus et al., 2017; Fukui et al., 2018; Cojocariu et al., 2020; Sugiyama and Shiotani, 2020; Lingpeng et al., 2021). Accumulating evidence indicates that ECs play a crucial role in the pathogenesis of ELS-induced IBS (Bian et al., 2011; Chow et al., 2019; Qin et al., 2019a; Wong et al., 2019) as ECs bidirectionally (top-down or down-top) mediated signaling transmission of the brain–gut–microbiota axis (Stasi et al., 2012). The potential role of ECs in ELS-induced IBS is summarized in Figure 5.

## Links Between Enterochromaffin Cells, Corticotropin-Releasing Hormone, and Early Life Stress-Induced Irritable Bowel Syndrome

Early life (<3 years of age) represents a particularly important developmental period for the gut microbiota (Osadchiy et al., 2019), CNS (Codagnone et al., 2019), intestinal barriers (Selma-Royo et al., 2020), ENS (Joly et al., 2020), and immune, endocrine, metabolic, and other host developmental pathways (Robertson et al., 2019). This period is of key importance, and once disturbed, may have long-lasting effects (Codagnone et al., 2019). Stress, in particular, can significantly influence the brain–gut–microbiota axis at all stages of life (Cryan et al., 2019) through the hypothalamic–pituitary–adrenal (HPA) axis (Farzi et al., 2018). The HPA axis is the major neuroendocrine system in the body that controls diverse body processes in response to stress and closely interacts with the gut microbiota (Farzi et al., 2018).

Evidence has shown that ELS leads to HPA axis activation (Wong et al., 2019). This hyperactivity is related to increased CRH signaling and impaired glucocorticoid receptor-mediated negative feedback (van Bodegom et al., 2017). The initiation of HPA axis activation is CRH released from the hypothalamus (Vale et al., 1981), which stimulates the release of adrenocorticotropic hormone (ACTH) from the pituitary. There are two CRH receptors (CRH-R): CRH-R1 and CRH-R2. In the brain, CRH-R1 is highly expressed, whereas in peripheral tissues, it is unremarkably expressed. In contrast, CRH-R2 is less expressed in the brain and more highly expressed in peripheral tissues (Ketchesin et al., 2017). Brain sites of CRH are known to alter gut motility, encompassing the locus coeruleus complex, paraventricular nucleus of the hypothalamus, and the dorsal motor nucleus, while those modulating visceral pain are localized in the hippocampus and central amygdala. Brain CRH actions are mediated through the autonomic nervous system (decreased gastric vagal and increased sacral parasympathetic and sympathetic activities) (Tache et al., 2018). Importantly, CRH is a critical neuropeptide in the gut–brain axis and can regulate various biological activities (Wei et al., 2020). First, neuropeptides are components of the autonomic nervous system and act locally at peripheral sites as neurotransmitters. Second, neuropeptides act on central regulatory centers as neuromodulators. Finally, neuropeptides can reach the immune system, peripheral vessels, organs, and glands through the circulatory system as a neurohormone and hormone (Lotti et al., 2014). Accumulating evidence has shown that CRH release causes bowel dysfunction through multiple pathways, either through the HPA axis, the autonomic nervous system, or directly on the bowel itself (Chang et al., 2014). Moreover, CRH influences the composition of the gut microbiota (de Punder and Pruimboom, 2015). Activation of both brain and peripheral CRH-R1 reduces the pain threshold for colonic distention and increases colonic motility. Furthermore, activation of the corticotropin releasing factor receptor 1 (CRF-R1) signaling pathway has been implicated in the development of anxiety-like behaviors (Taché et al., 2004). Moreover, intraperitoneal injection of corticotropin releasing factor (CRF), subcutaneous injection of lipopolysaccharide, and repeated water avoidance stress (WAS) all lead to visceral allodynia and increased colonic permeability, which is CRH-R1 dependent, while activation of CRH-R2 inhibits CRH-R1-triggered responses (Nozu et al., 2018). Together, these results indicate that CRH acting on CRH-R1 mediates visceral hypersensitivity, colonic permeability, and intestinal motility, while CRH acting on CRH-R2 has the opposite effect (Tache et al., 2018).

CRH-R1 is expressed in intestinal myenteric neurons (Fukudo, 2007), MCs (D’Costa et al., 2019), and dendritic cells (Lee et al., 2009), and is also expressed in ECs (Wu et al., 2011). Using *in vitro* experiments, Wu et al. (2011) revealed that exposure of BON cells to CRH for 24 h upregulated TPH1 mRNA levels and activated CRH-R1-dependent pathways leading to 5-HT synthesis and release. Furthermore, CRF receptor antagonists have been proven to be useful in attenuating intestinal ECs hyperplasia (Qin et al., 2019a). These results suggest that ECs may be involved in ELS-induced IBS. In a recent study using an established maternal separation (MS) animal model and intestinal organoid culture, Wong et al. (2019) demonstrated that ELS led to increased CRF and induced elevated ISC expansion and their differentiation toward secretory lineages, including ECs and Paneth cells, leading to ECs hyperplasia, increased 5-HT production, visceral hyperalgesia, and intestinal motility. In addition, results from an experimental study suggested that ELS induced ECs hyperplasia in the gut of adult animals (Bian et al., 2011). TPH1 expression was markedly upregulated in the MS animal model and was associated with visceral pain (Distrutti et al., 2013). The increased synthesis of 5-HT by ECs or disrupted 5-HT uptake resulted in increased bioavailability of 5-HT, which is involved in the generation of IBS symptoms, such as intestinal motility alteration, increased secretion, visceral hypersensitivity, and increased permeability (Qin et al., 2019a,b). Additionally, higher 5-HT content, higher TPH1 expression, and lower SERT mRNA were detected in both children and adults with IBS, compared with that of the controls (Faure et al., 2010; Yu et al., 2016; Fu R. et al., 2019). Furthermore, TPH1 inhibition and colonic 5-HT content was significantly reduced, and visceral hyperalgesia was alleviated (Shi et al., 2018). Together, these results indicate that ELS-induced CRH elevation and ECs increase. CRH and ECs may play an important role in ELS-induced IBS (Figure 5).

## Links Between Enterochromaffin Cells, Mast Cells, and Early Life Stress-Induced Irritable Bowel Syndrome

The essential role of MCs in the occurrence and development of IBS has been well recognized (Xu et al., 2017; Miura et al., 2020). In both IBS patients and experimental animals, MCs were reported to be significantly increased in the intestine (Bednarska et al., 2017; Xu et al., 2017; McClain et al., 2020; Miura et al., 2020). The increased MCs were shown to be associated with various IBS phenotypes, including damage to the intestinal barrier (Zhang et al., 2016), dysfunction of intestinal motility (Camilleri et al., 2017), low-grade inflammation (Uranga et al., 2020), visceral hypersensitivities (Kamphuis et al., 2020), and translocations of the gut microbiota (Bednarska et al., 2017). Moreover, the number of MCs was significantly related to the severity of symptoms in both IBS patients and the IBS experimental animal model (Akbar et al., 2008; Kamphuis et al., 2020). The symptoms observed in IBS patients and animal models were attributed to the fact that MCs contain multiple mediators of paracrine signaling, including histamine, serine proteases, chymase, tryptase, prostaglandin, and 5-HT (Galli et al., 2020; Wood, 2020). These diverse mediators are released from MCs through degranulation, which can be triggered by multiple neuropeptides, neurotransmitters, hormones, and bacterial secretions, such as SP, VIP, calcitonin gene-related peptide (CGRP), CRH, 5-HT, and quorum-sensing molecules (QSMs) (Wang et al., 2013; D’Costa et al., 2019; Guilarte et al., 2020; Wei et al., 2020; Wood, 2020). Moreover, increased SP, VIP, CGRP, CRH, and 5-HT were observed in IBS patients and experimental IBS animals (Keszthelyi et al., 2013; Yu et al., 2016; Ceuleers et al., 2018; Fu R. et al., 2019; Guilarte et al., 2020).

MCs are immune cells with a widespread distribution, including the digestive tracts surrounding blood vessels, neurons, or nerve fibers, primarily in the intestinal lamina propria (Uranga et al., 2020; Wood, 2020). As previously mentioned, ECs and MCs are neighbors, as ECs are located on the epithelial layer (Mawe and Hoffman, 2013), and MCs are mainly located in the lamina propria (Zhang et al., 2016; Uranga et al., 2020). ECs express histamine receptors 1 and 3 (Pfanzagl et al., 2019), while MCs express 5-HT_1A_R (Schwörer et al., 1992), indicating that a possible connection exists between these two cells. A clinical study showed that ECs number and 5-HT content were significantly increased in IBS patients, particularly in IBS-D, compared to that of the control. 5-HT content was correlated with MCs counts and the severity of abdominal pain (Martínez et al., 2013). The results indicate that ECs contribute to the development of abdominal pain in IBS, likely through MCs activation (Lobo et al., 2017). A systematic review revealed MCs and ECs both increased in IBS patients, despite the limited intrinsic heterogeneity of IBS and a lack of standardization in study design (Martin-Viñas and Quigley, 2016). However, Lee et al. (2008) found significantly increased MCs in irritable bowel syndrome-diarrhoea (IBS-D) without ECs number alterations in non-post-infectious IBS patients, which is likely owing to the small sample size. Together, the evidence supports the viewpoint that both MCs and ECs numbers are associated with IBS, as well as the severity of IBS symptoms.

Mechanistically, ECs and MCs might have a synergistic effect on the pathogenesis of ELS-induced IBS. ELS leads to the release of CRH, alone or together with other peptides such as SP, which then stimulate MCs to secrete proinflammatory molecules (D’Costa et al., 2019). Some of these molecules may cross-talk with ECs. First, histamine acts on histamine receptor-mediated visceral hypersensitivity (Balemans et al., 2019). ECs have histamine receptors (Pfanzagl et al., 2019), and ELS induces ECs hyperplasia, leading to visceral hypersensitivity (Wong et al., 2019). Second, mediators released from the intestinal mucosa of IBS patients, such as histamine, 5-HT, and proteases, can activate submucosal neurons. However, this activation is inhibited by histamine receptor antagonists, 5-HT_3_R antagonists, and protease inhibition (Buhner et al., 2009). A recent study also revealed that colon biopsies from patients with IBS-D had increased levels of prostaglandin E2. Intracolonic infusions of rats with IBS-D biopsy supernatants increased visceral hypersensitivity, which was associated with a significant increase in prostaglandin E2, histamine, and tryptase in the colonic mucosa (Grabauskas et al., 2020). Third, patients with IBS had increased bacterial translocation compared with that of the controls, indicating increased intestinal permeability. The mechanisms of increased translocation include MCs tryptase and VIP (Bednarska et al., 2017). Finally, 5-HT acted as an interkingdom signaling molecule via quorum sensing, stimulated the production of bacterial virulence factors, and increased biofilm formation *in vitro* and *in vivo* in a novel mouse infection model (Knecht et al., 2016). Meanwhile, MCs have receptors for gram-positive QSMs, and trigger degranulation upon activation by QSMs, which inhibits bacterial growth and prevents biofilm formation (Pundir et al., 2019). Therefore, ECs and MCs may play a crucial role in the pathogenesis of ELS-induced IBS (Figure 5).

## Links Between Enterochromaffin Cells, Nerve Growth Factor, and Early Life Stress-Induced Irritable Bowel Syndrome

The critical role of NGF in the pathogenesis of IBS is well established (Coelho et al., 2019). In colonic biopsies obtained from adult and pediatric patients with IBS, NGF levels were found to be upregulated (Willot et al., 2012; Xu et al., 2017). Another study suggested that NGF is closely related to IBS with constipation (IBS-C) (Lee et al., 2020). Furthermore, NGF expression is positively correlated with disease severity and anxiety, and is negatively associated with the threshold of visceral sensitivity (Xu et al., 2017). Furthermore, increased NGF levels have also been observed in rodents exposed to ELS (Chow et al., 2019; Wong et al., 2019). Moreover, MS promotes hyperplasia in both MCs and synaptogenesis in rats, whereas treatment of pups with anti-NGF antibodies abolishes this effect (Barreau et al., 2008). In further studies on MS of rodents, Wong et al. (2019) revealed that NGF elevation directly targeted ISC with trans-activating Wnt/β-catenin signaling and promoted their expansion and differentiation to intestinal ECs hyperplasia, resulting in visceral hypersensitivity. These important findings indicate that, in the initiation and development of IBS, proliferation and differentiation of ISC may be disrupted, and ECs hyperplasia may be induced in newborn animals who experience ELS. However, there is a lack of clinical evidence indicating that patients with IBS who experience ELS present high levels of NGF and ECs hyperplasia. One study revealed that nerve fiber outgrowth and staining density of NGF were increased in the intestinal mucosa of patients with IBS (Dothel et al., 2015), however, considerable advancement is required for its clinical application. Therefore, the effects of ECs and NGF on the pathogenesis of ELS-induced IBS require further clinical studies.

## Links Between Enterochromaffin Cells, Bile Acids, and Early Life Stress-Induced Irritable Bowel Syndrome

Bile acids are known to play a pivotal role in the pathological development of IBS, particularly IBS-D (Aziz et al., 2015; Zhao et al., 2020). In addition, bile acid level is associated with the severity of IBS symptoms. IBS patients with moderate or severe bile acid diarrhea are significantly less physically active than those with mild bile acid diarrhea (Aziz et al., 2015). In addition, increased colonic bile acid exposure results in more frequent bowel movements and accelerated colonic transit time in patients with IBS compared to that in controls (Bajor et al., 2015). Furthermore, in rats, deoxycholic acid (DCA) directly excited spinal afferents and, to a lesser extent, indirectly via mucosal 5-HT release, while DCA increased vagal afferent firing in the proximal colon via 5-HT release. Intraluminal DCA caused an increase in ECs density, which was greater in the proximal colon than in the distal colon. These results suggest that ECs partly mediate signaling from DCA to spinal afferents, whereas they mediate signaling from DCA to the vagal afferent (Yu et al., 2019). One of the potential mechanisms is that DCA, a SBA, can act on TGR5 of ECs and promote 5-HT synthesis (Lund et al., 2018). TGR5, a membrane receptor of bile acid receptors, is widely expressed throughout the small intestine and colon, and participates in the modulation of intestinal functions (de Aguiar Vallim et al., 2013; Martinot et al., 2017; Wei et al., 2021). One study detected TGR5 expression in ECs in the colon, however, not in the small intestine (Lund et al., 2018). Colonic mucosal TGR5 protein expression is correlated with the severity of symptoms in IBS-D patients (Wei et al., 2021). These results indicate that TGR5-ECs-5-HT signaling may play a role in the pathophysiology of IBS.

In addition, evidence from the animal experiments confirmed that HFD rats exhibited significantly increased levels of cholic acid and DCA in intestinal contents accompanied by upregulation of TPH1 protein expression compared with that of the control. Moreover, GI transit and small intestinal 5-HT concentrations were higher in HFD-fed rats than in control rats. When the rats received fecal microbiota transplantation treatment from the control, the GI transit, small intestinal 5-HT concentration, and TPH1 expression were decreased, accompanied by decreased cholic acid and DCA (Sun et al., 2018). Moreover, ELS-induced increased intestinal permeability, visceral sensitivity, and bile acid malabsorption were detected. Bile acid malabsorption was reported to be due to alteration of the intestinal microbiota (Riba et al., 2018). However, neither ECs nor TPH1 expression was detected. Considering previous reports, it could be postulated that ELS leads to ECs hyperplasia (Wong et al., 2019) and bile acid malabsorption (Riba et al., 2018). Therefore, ECs and bile acids may be involved in the pathophysiology of ELS-induced IBS (Figure 5). Additional studies are required to investigate this further.

## Links Between Enterochromaffin Cells, Short-Chain Fatty Acids, and Early Life Stress-Induced Irritable Bowel Syndrome

SCFAs are produced by gut microbial fermentation of indigestible dietary fiber and serve as energy sources and natural ligands for a group of orphan GPCRs, which play an important role in metabolism and immunity (Tian et al., 2020). An increasing number of studies have shown that SCFAs, primarily acetate, propionate, and butyrate, might play a role in the pathophysiology of IBS. Reduced SCFA levels have been reported in IBS-C patients compared to that in unsubtyped IBS and IBS-D patients (Gargari et al., 2018). Recently, a systematic review and meta-analysis showed that propionate and butyrate levels were reduced in IBS-C patients, whereas butyrate was increased in IBS-D patients compared to that in healthy controls (Sun et al., 2019). Furthermore, both clinical and animal experiments suggested that, compared with their corresponding controls, IBS-D patients and MS rats showed a higher abundance of SCFA-producing *Fusobacterium*. Moreover, the abundance of *Fusobacterium* was positively correlated with the degree of visceral hypersensitivity (Gu et al., 2020). This indicates that the elevation of SCFAs is associated with visceral hypersensitivity. Butyrate is known to be the most important metabolite and functions as a major energy source for colonocytes by directly affecting the growth and differentiation of colonocytes. Furthermore, butyrate exerts various physiological effects, such as enhancement of intestinal barrier function and mucosal immunity (Fu X. et al., 2019). Results from the WAS model suggested that butyrate had a stimulating effect on longitudinal muscle at low concentrations (1 mM to 10 mM), while exhibiting an inhibitory effect at high concentrations (30 mM) (Yuan et al., 2020). Moreover, Akiba et al. (2017) suggested that luminal FFAR2 agonists stimulate ECs to release 5-HT, which enhances mucosal defenses in the rat duodenum. However, excessive 5-HT release with high luminal concentrations of SCFAs injures the mucosa by decreasing mucosal blood flow, which is likely implicated in the 5-HT-related mechanism. Small intestinal bacterial overgrowth (SIBO) has been hypothesized to generate excess SCFAs in the foregut and promote ECs to release unconscionable 5-HT (Akiba et al., 2017), and has been recognized as a hallmark of IBS (Shah et al., 2020). Notably, as previously described, ECs express SCFA receptors, including FFAR2 and FFAR3 (Nøhr et al., 2013; Pfanzagl et al., 2019). SCFAs upregulate TPH1 expression, thereby promoting ECs to produce 5-HT by acting on their SCFA receptors (Reigstad et al., 2015). Moreover, treatment with rifaximin, an antibiotic that is not absorbed systemically and is currently approved for the treatment of IBS (Brenner and Sayuk, 2020), improves SIBO and ameliorates abdominal symptoms of IBS (Li et al., 2020). Taken together, these results indicate that a normal concentration of SCFAs, particularly butyrate, is beneficial to intestinal barrier function. However, high SCFA concentrations may be harmful. Therefore, the increased butyrate observed in IBS-D patients (Gargari et al., 2018; Sun et al., 2019) may affect the intestinal barrier, as both increased intestinal permeability and visceral hypersensitivity are features of IBS.

Conversely, SCFAs were reduced in IBS-C patients, including propionate and butyrate (Gargari et al., 2018; Sun et al., 2019), which indicated that low 5-HT levels were produced by ECs. Indeed, IBS-C patients presented low 5-HT levels in the gut and were thus treated with 5-HT_3_R and 5-HT_4_R agonists (Binienda et al., 2018). In parallel, certain IBS-C patients also showed increased intestinal permeability and visceral sensitivity (Annaházi et al., 2013). It has been documented that reduced SCFAs levels, particularly butyrate, may have adverse effects on epithelial barrier integrity and energy homeostasis (Morris et al., 2017). 5-HT is involved in the regulation of mucosal homeostasis by promoting epithelial growth (Greig et al., 2016). This might explain why IBS-C patients with reduced SCFAs also manifested increased permeability and visceral sensitivity, similar to IBS-D patients who had a relatively higher SCFAs level. Therefore, either weakened or enhanced SCFAs-ECs signaling may be implicated in the pathogenesis of IBS. This viewpoint was supported by animal experiments. Rats that underwent chronic WAS caused visceral hypersensitivity and decreased occludin expression in the colon, accompanied by reduced butyrate, as well as reduced abundance of several butyrate-producing bacteria, such as *Lachnospiraceae*. Supplementation of *Roseburia*, a species belonging to *Lachnospiraceae*, to WAS rats significantly increased cecal butyrate content, alleviated visceral hypersensitivity, and prevented a decrease in occludin expression (Zhang et al., 2019). Similarly, another animal experiment indicated that WAS induced GI hypermotility, and fecal SCFAs were decreased significantly (Yuan et al., 2020). In addition, the level of 5-HT in colonic tissue also decreased when animals were exposed to WAS (Shi et al., 2015). These results indicate that chronic WAS leads to visceral hypersensitivity and increased intestinal permeability, which is associated with reduced SCFAs (particularly butyrate) and 5-HT.

Moreover, butyric acid levels were significantly lower in the MS group than in the non-separation group (Egerton et al., 2020). In addition, Egerton et al. (2020) found that MS animals had significantly lower ratios of SCFA producers, including *Caldicoprobacteraceae*, *Streptococcaceae*, *Rothia*, *Lachnospiraceae_NC2004_group*, and *Ruminococcus_2*. Additionally, animal exposure to prolonged restraint significantly reduced SCFAs, and *Lactobacillus* in the gut was significantly reduced, which was directly correlated with propionic acid (Maltz et al., 2018). However, these studies did not consider ECs or 5-HT. As previously mentioned, MS induces ECs hyperplasia and a high 5-HT level (Wong et al., 2019). This resulted in conflicting results. Reduced SCFAs and an increased number of ECs coexist in maternally separated animals, however, causality is undetermined. If reduced SCFAs is the cause, then it is difficult to understand why ECs are hyperplastic. SCFAs have been shown to upregulate TPH1 expression but do not change the number of ECs (Reigstad et al., 2015). Furthermore, a previous study showed reduced SCFAs with reduced 5-HT (Shi et al., 2015). Consequently, the cause tends to the latter, that is to say, the increased number of ECs caused reduced SCFAs or ECs hyperplasia that appeared first and then reduced SCFAs. However, the underlying mechanism is unclear. The possible cause is that intraluminal excessive 5-HT released from ECs may inhibit the abundance of SCFA-producing microbiota, likely in a manner of negative feedback. Recently, two studies have suggested the effects of 5-HT on intestinal bacteria. One study conducted by Kumar et al. (2020) revealed that 5-HT modulated the virulence of enteric pathogens, and another study by Fung et al. (2019) showed that 5-HT modulated bacterial colonization in the gut. The significant implication of these two studies is that they elucidate the mechanism of action of 5-HT on bacteria. Accordingly, it is interesting to investigate the role of the ECs-5-HT-SCFAs signal in ELS-induced IBS (Figure 5).

## Future Perspectives

ECs sense various stimuli, such as nutritional, chemical, odorous, mechanical, hypoxic, and bacterial metabolites, including SCFAs and SBA, indicating the important role of ECs in maintaining intestinal homeostasis. Simultaneously, because of the peculiarity of their location in the gut, ECs can transmit luminal signals to neighboring cells and neurons, and even to the CNS.

Despite considerable efforts having been made, the pathogenesis of IBS remains unclear. However, an emerging number of studies have shown the crucial role of ECs in the pathogenesis of ELS-induced IBS. IBS is a disorder of dysfunction of the brain–gut–microbiota, while ECs link microbiota, enteroendocrine cells, intestinal epithelial cells, enteric immune cells, the ENS, and the CNS. Therefore, either weakened or enhanced function of ECs may affect the brain–gut–microbiota axis, and thus play an important role in the pathogenesis of IBS. ECs-5-HT dysfunction is associated with IBS symptoms, including increased intestinal permeability, secretion, intestinal mobility, visceral hypersensitivity, low-grade inflammation, and altered intestinal microbiota. Furthermore, ECs have a close relationship with CRF, NGF, MCs, SCFAs, and bile acids, which are involved in the pathogenesis of IBS.

By exploring unclear mechanisms and undiscovered actions of ECs in the brain–gut–microbiota axis in the pathogenesis of ELS-induced IBS, ECs may provide new insights into the potential therapeutic targets and diagnostic markers in the treatment and management of IBS. Manipulating ECs and maintaining the ECs-5-HT signaling balance may be helpful in the prevention and treatment of IBS, particularly in those who had previously suffered ELS.

## Author Contributions

MJ contributed to the conceptualization, funding acquisition, and writing – review and editing. ET contributed to the validation, formal analysis, investigation, writing – original draft, writing – review and editing, and visualization. ZZ contributed to the writing – review and editing, and visualization. CH, GL, BC, and RG contributed to the validation, formal analysis, and writing – review and editing. MF contributed to the validation, formal analysis, writing – review and editing, and supervision. All authors contributed to the article and approved the submitted version.

## Conflict of Interest

The authors declare that the research was conducted in the absence of any commercial or financial relationships that could be construed as a potential conflict of interest.

## Publisher’s Note

All claims expressed in this article are solely those of the authors and do not necessarily represent those of their affiliated organizations, or those of the publisher, the editors and the reviewers. Any product that may be evaluated in this article, or claim that may be made by its manufacturer, is not guaranteed or endorsed by the publisher.

## Footnotes

1
http://www.genomatix.de/
